# Modeling and Quantitative Analysis of GNSS/INS Deep Integration Tracking Loops in High Dynamics

**DOI:** 10.3390/mi8090272

**Published:** 2017-09-08

**Authors:** Yalong Ban, Xiaoji Niu, Tisheng Zhang, Quan Zhang, Jingnan Liu

**Affiliations:** 1 GNSS Research Center, Wuhan University, 129 Luoyu Road, Wuhan 430079, China; ylban@whu.edu.cn (Y.B.); xjniu@whu.edu.cn (X.N.); zhangquan@whu.edu.cn (Q.Z.); jnliu@whu.edu.cn (J.L.); 2 Collaborative Innovation Center of Geospatial Technology, 129 Luoyu Road, Wuhan 430079, China

**Keywords:** global navigation satellite systems (GNSS)/inertial navigation system (INS) deep integration, high dynamics, error propagation model, quantitative analysis

## Abstract

To meet the requirements of global navigation satellite systems (GNSS) precision applications in high dynamics, this paper describes a study on the carrier phase tracking technology of the GNSS/inertial navigation system (INS) deep integration system. The error propagation models of INS-aided carrier tracking loops are modeled in detail in high dynamics. Additionally, quantitative analysis of carrier phase tracking errors caused by INS error sources is carried out under the uniform high dynamic linear acceleration motion of 100 g. Results show that the major INS error sources, affecting the carrier phase tracking accuracy in high dynamics, include initial attitude errors, accelerometer scale factors, gyro noise and gyro g-sensitivity errors. The initial attitude errors are usually combined with the receiver acceleration to impact the tracking loop performance, which can easily cause the failure of carrier phase tracking. The main INS error factors vary with the vehicle motion direction and the relative position of the receiver and the satellites. The analysis results also indicate that the low-cost micro-electro mechanical system (MEMS) inertial measurement units (IMU) has the ability to maintain GNSS carrier phase tracking in high dynamics.

## 1. Introduction

### 1.1. Previous Work

High dynamics is usually interpreted as high values for velocity and its derivatives. The Jet Propulsion Laboratory (JPL) has defined two high dynamic trajectories: one is linear acceleration of no less than 50 g, and the other is circular motion with a period of 6–8 s and with radial acceleration of 50 g [1,2]. The high dynamics of vehicles bring serious impact to the stability of global navigation satellite system (GNSS) signal carrier phase tracking, leading to the failure of GNSS precise positioning [3,4]. The inertial navigation system (INS) has a superior dynamic characteristic, which is highly complementary to GNSS [5,6]. In the GNSS/INS deep integration, the impact of the dynamics on the tracking loops can be mitigated by INS aiding, which is helpful for the improvement of the loop performance [7,8,9].

**Figure 1:**
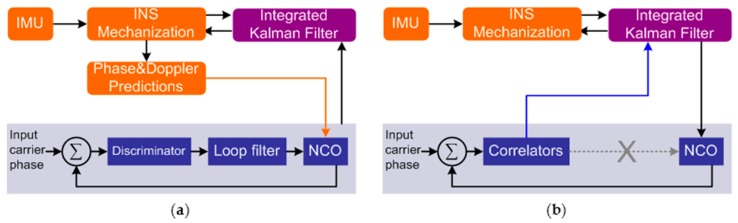
Two architectures of global navigation satellite systems (GNSS)/inertial navigation system (INS) deep integration. (**a**) Scalar-based architecture; (**b**) vector-based architecture. NCO, numerically-controlled oscillator.

Based on the type of tracking loops used in receivers, the GNSS/INS deep integration can be implemented in two different ways [10], shown in Figure 1, respectively named: scalar-based architecture [11,12] and vector-based architecture [13,14]. The scalar-based architecture keeps the individual traditional tracking loops and is aided by INS, while in the vector-based architecture, the traditional individual tracking loops are eliminated, and it can make full use of the available information and get better signal sensitivity. However, since the deeply-coupled Kalman filter output accuracy is insufficient for the carrier phase, the individual carrier phase tracking loops still need to be used in the vector-based architecture [15]. Therefore, the study of the carrier phase in deep integration was carried out on individual tracking loops.

In a deeply-coupled system, the accuracy of the INS-aided data is critical to the tracking loops, especially for the low-cost inertial measurement unit (IMU), as they have poor error characteristics. Therefore, an effective quantitative analysis method is urgently needed to design and implement deep integration based on the model of INS-aided tracking loops. To the authors’ knowledge, the existing models do not fully consider INS error sources and cannot correctly reflect the error transformation of INS in the tracking loop [16,17,18]. Hence, they cannot be directly used for quantitative analysis of the impact of the INS on the receiver tracking loop, especially for high dynamics. The authors’ previous work has made an effort on the INS-aided branch modeling in low dynamics [19,20]. The transfer function between the error sources and the phase-locked loops’ (PLLs) tracking error has established, and the negative effects of the inertial aiding information from different grades of INS in low dynamics have been quantitatively assessed, which were not applied in high dynamics.

### 1.2. Objectives

**Figure 2:**
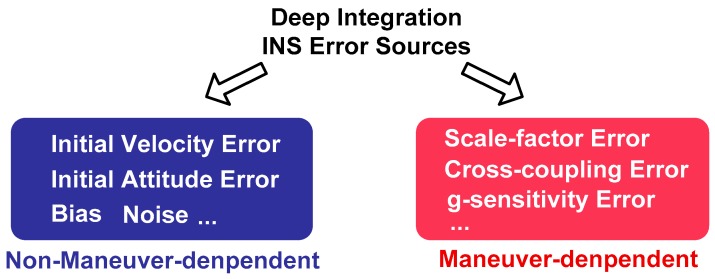
The classification of the INS error sources in the GNSS/INS deep integration.

In practice, the performance of the INS is very dependent on the motion of the vehicle [16]. The large angular rate and acceleration output from the inertial sensors will cause multiple error sources when the host vehicle maneuvers, such as the scale-factor error, the cross-coupling error, the gyro g-sensitivity error, etc. Meanwhile, the impact of INS initial error, bias and noise exist whether the vehicle is stationary or maneuvering. Therefore, the INS error sources can be divided into maneuver-dependent error terms and non-maneuver-dependent error terms, presented in Figure 2. Previous work mainly evaluates the impact of the non-maneuver-dependent errors in low dynamics [19,20]. However, the effect of maneuver-dependent errors may become prominent and affect the tracking performance seriously in high dynamics.

In this paper, a quantitative analysis method is proposed to evaluate the impact of the maneuver-dependent and non-maneuver-dependent errors of different grades of IMU on the receiver PLLs. Compared with our previous work, the main contribution is that modeling and quantitative analysis are carried out in high dynamics [20]. The model and quantitative analysis will be a reference and guidance when designing carrier phase tracking loops in the GNSS/INS deep integrated system for precise positioning applications in high dynamics.

The rest of this paper is organized as follows: Firstly, it presents the detailed transfer relation of the micro-electro mechanical system (MEMS) and tactical INS error sources and the carrier phase tracking errors in the Laplace domain. Then, it performs the time domain quantitative analysis of tracking errors caused by the maneuver-dependent and non-maneuver-dependent errors, respectively. Finally, the conclusions of this study are given.

## 2. Methodology

**Figure 3:**
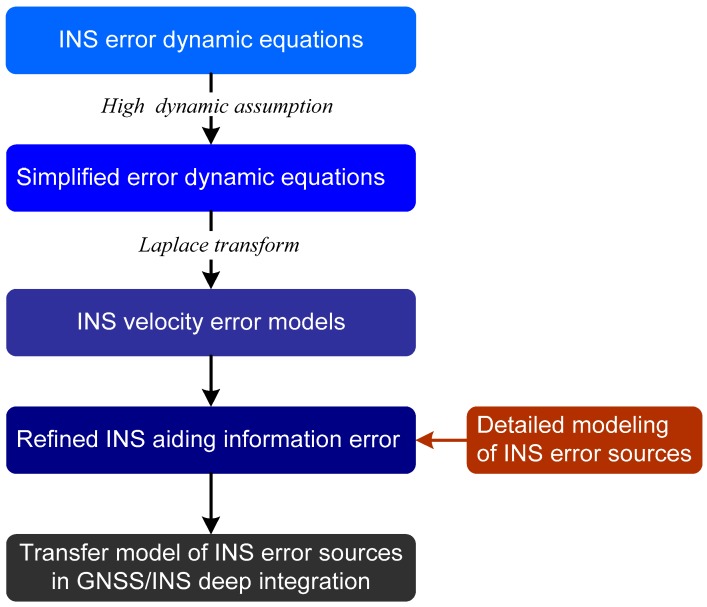
The modeling process of the relationship of INS error sources and the carrier phase tracking error in the GNSS/INS deep integration.

It is necessary to establish the transfer model of INS error sources in the GNSS/INS deep integration before quantitatively analyzing its impact on carrier phase tracking errors. The process of the modeling is shown in Figure 3. It starts with the INS error dynamic equations, and some reasonable simplifications should be made firstly based on the assumption of uniform high dynamic linear acceleration motion. Then, the INS velocity error models can be obtained by solving the simplified INS error dynamic equations according to the Laplace transform. After that, the INS-aided information error is calculated based on the principle of INS aiding [19], and the refined model can be achieved combined with the detailed modeling of INS error sources. Finally, substituting the INS-aided information error model into the transfer function of INS aided PLLs [20], the models reflecting the relationship of INS error sources and the tracking error can be established.

### 2.1. Error Dynamic Solutions of INS

The propagation of the INS errors can be represented as a set of difference equations, which are derived from the system equations by taking partial derivatives [21,22]. Some reasonable simplifications and assumptions can be achieved based on the peculiarity of the research objects [20].

(a) It is assumed that the vehicle is in uniform high dynamic linear acceleration motion (within 100 g acceleration and 1000 m/s velocity);
(b) The minor terms (e.g., terms contain the reciprocal of the Earth radius parameters) are ignored as they have little effect on the navigation errors;
(c) Assume that the sensor selection of each axis is the same, and it can be considered that the sensor error characteristics in the n-frame are the same as in the b-frame, because the rotation matrix from the b-frame to the n-frame is an identity and orthogonal matrix;
(d) The impact of position error can be ignored after simplification because the position errors do not affect the velocity errors and attitude errors in the analysis.

Then, the simplified error dynamic equations in the navigation frame (n-frame, north-east-down) of MEMS and tactical INS can be expressed as follows.

MEMS case:

(1) $$\left\{ \begin{array}{l} \delta {\dot{v}}_{N}=-f_{D}\phi_{pitch}+f_{E}\phi_{yaw}+\delta f_{N}\\ \delta {\dot{v}}_{E}=f_{D}\phi_{roll}-f_{N}\phi_{yaw}+\delta f_{E}\\ \delta {\dot{v}}_{D}=-f_{E}\phi_{roll}+f_{N}\phi_{pitch}+\delta f_{D}\\ {\dot{\phi }}_{roll}=-\delta \omega_{N}\\ {\dot{\phi }}_{pitch}=-\delta \omega_{E}\\ {\dot{\phi }}_{yaw}=-\delta \omega_{D} \end{array}\right.$$

Tactical case:

(2) $$\left\{ \begin{array}{l} \delta {\dot{v}}_{N}=-f_{D}\phi_{pitch}+f_{E}\phi_{yaw}+\delta f_{N}\\ \delta {\dot{v}}_{E}=f_{D}\phi_{roll}-f_{N}\phi_{yaw}+\delta f_{E}\\ \delta {\dot{v}}_{D}=-f_{E}\phi_{roll}+f_{N}\phi_{pitch}+\delta f_{D}\\ {\dot{\phi }}_{roll}=\frac{v_{N}}{R_{M}+h}\phi_{yaw}-\delta \omega_{N}\\ {\dot{\phi }}_{pitch}=\left(\omega_{e}\cos\varphi +\frac{v_{E}}{R_{N}+h}\right)\phi_{yaw}-\delta \omega_{E}\\ {\dot{\phi }}_{yaw}=-\delta \omega_{D} \end{array}\right.$$

All symbols in Equations (1) and (2) are defined as follows: operator *δ* means the error of something; δν and ϕ are the velocity and attitude error in the *n*-frame, respectively; δf is the error for accelerometers; and δω is the error of gyros. φ is the geographical latitude; $R_{M}$, $R_{N}$ are the radii of curvature in the meridian and prime vertical; *h* is the ellipsoidal height.

Then, the simplified error dynamics can be expressed in matrix form as follows:

(3) $$\dot{x}(t)=F(t)x(t)+w(t)$$

and:

$$F_{MEMS}=\left(\begin{array}{cccccc} 0 & 0 & 0 & 0 & -f_{D} & f_{E}\\ 0 & 0 & 0 & f_{D} & 0 & -f_{N}\\ 0 & 0 & 0 & -f_{E} & f_{N} & 0\\ 0 & 0 & 0 & 0 & 0 & 0\\ 0 & 0 & 0 & 0 & 0 & 0\\ 0 & 0 & 0 & 0 & 0 & 0 \end{array}\right)\;F_{T\mathrm{ac}tical}=\left(\begin{array}{cccccc} 0 & 0 & 0 & 0 & -f_{D} & f_{E}\\ 0 & 0 & 0 & f_{D} & 0 & -f_{N}\\ 0 & 0 & 0 & -f_{E} & f_{N} & 0\\ 0 & 0 & 0 & 0 & 0 & v_{N}/R\\ 0 & 0 & 0 & 0 & 0 & \omega_{e}\cos\varphi +v_{E}/R\\ 0 & 0 & 0 & 0 & 0 & 0 \end{array}\right)$$

Here, the elements of F(t) are constant or changing slowly in a short time (as the vehicle is in uniform linear acceleration motion), so the Laplace transform can be finished as [16,23,24]:

(4) $$sx(s)=F\cdot x(s)+x(0)+w(s)\Rightarrow x(s)={\left(sI-F\right)}^{-1}[x(0)+w(s)]$$

where I is the identity matrix and x(0) is the initial value of x(t). Then, the velocity error of MEMS and tactical INS in the Laplace domain can be derived from Equation (4).

MEMS case:

(5) $$\left\{ \begin{array}{l} \delta v_{N}(s)=\frac{1}{s}\delta v_{N}(0)-\frac{f_{D}}{s^{2}}\phi_{pitch}\left(0\right)+\frac{f_{E}}{s^{2}}\phi_{yaw}\left(0\right)+\frac{1}{s}\delta f_{N}(s)+\frac{f_{D}}{s^{2}}\delta \omega_{E}(s)-\frac{f_{E}}{s^{2}}\delta \omega_{D}\left(s\right)\\ \delta v_{E}(s)=\frac{1}{s}\delta v_{E}(0)+\frac{f_{D}}{s^{2}}\phi_{roll}\left(0\right)-\frac{f_{N}}{s^{2}}\phi_{yaw}\left(0\right)+\frac{1}{s}\delta f_{E}(s)-\frac{f_{D}}{s^{2}}\delta \omega_{N}(s)+\frac{f_{N}}{s^{2}}\delta \omega_{D}\left(s\right)\\ \delta v_{D}(s)=\frac{1}{s}\delta v_{D}(0)-\frac{f_{E}}{s^{2}}\phi_{roll}\left(0\right)+\frac{f_{N}}{s^{2}}\phi_{pitch}\left(0\right)+\frac{1}{s}\delta f_{D}(s)+\frac{f_{E}}{s^{2}}\delta \omega_{N}(s)-\frac{f_{N}}{s^{2}}\delta \omega_{E}(s) \end{array}\right.$$

Tactical case:

(6) $$\left\{ \begin{array}{ll} \delta v_{N}\left(s\right) & =\frac{1}{s}\delta v_{N}\left(0\right)-\frac{f_{D}}{s^{2}}\phi_{pitch}\left(0\right)+\frac{f_{E}s-\left(\omega_{e}\cos\varphi +\Lambda_{E}\right)f_{D}}{s^{3}}\cdot \phi_{yaw}\left(0\right)\\ & +\frac{1}{s}\delta f_{N}\left(s\right)+\frac{f_{D}}{s^{2}}\delta \omega_{E}\left(s\right)-\frac{f_{E}s-\left(\omega_{e}\cos\varphi +\Lambda_{E}\right)f_{D}}{s^{3}}\cdot \delta \omega_{D}\left(s\right)\\ \delta v_{E}\left(s\right) & =\frac{1}{s}\delta v_{E}\left(0\right)+\frac{f_{D}}{s^{2}}\phi_{roll}\left(0\right)-\frac{f_{N}s-\Lambda_{N}f_{D}}{s^{3}}\cdot \phi_{yaw}\left(0\right)\\ & +\frac{1}{s}\delta f_{E}\left(s\right)-\frac{f_{D}}{s^{2}}\delta \omega_{N}\left(s\right)+\frac{f_{N}s-\Lambda_{N}f_{D}}{s^{3}}\cdot \delta \omega_{D}\left(s\right)\\ \delta v_{D}\left(s\right) & =\frac{1}{s}\delta v_{D}\left(0\right)-\frac{f_{E}}{s^{2}}\phi_{roll}\left(0\right)+\frac{f_{N}}{s^{2}}\phi_{pitch}\left(0\right)+\frac{\left(\omega_{e}\cos\varphi +\Lambda_{E}\right)f_{N}-\Lambda_{N}f_{E}}{s^{3}}\cdot \phi_{yaw}\left(0\right)\\ & +\frac{1}{s}\delta f_{D}\left(s\right)+\frac{f_{E}}{s^{2}}\delta \omega_{N}\left(s\right)-\frac{f_{N}}{s^{2}}\delta \omega_{E}\left(s\right)-\frac{\left(\omega_{e}\cos\varphi +\Lambda_{E}\right)f_{N}-\Lambda_{N}f_{E}}{s^{3}}\cdot \delta \omega_{D}\left(s\right) \end{array}\right.$$

where $\Lambda_{N}=\frac{v_{N}}{R}$, $\Lambda_{E}=\frac{v_{E}}{R}$, $\delta f_{*}(s)$ is the accelerometer error, $\delta \omega_{*}(s)$ is the gyro error, $\delta v_{*}(0)$ is the initial velocity error and $\phi_{*}(0)$ is the initial attitude angle error. It should be noted that the initial errors in the GNSS/INS integration system are the residual errors right after the GNSS measurements update.

### 2.2. Detailed Modeling of the Error Sources in IMU

Equations (5) and (6) provide the velocity error solution of INS error dynamic equations. The initial errors (i.e., initial velocity error and initial attitude error) can be modeled as random-constant; however, the models of sensor errors (i.e., the accelerometer error and the gyro error) in the equations are not specific enough for error propagation analysis and should be further modeled [25]. In general, the accelerometer error and the gyro error can be respectively expressed in the time domain as [26]:

(7) $$\delta f\left(t\right)=\left[b_{c\_a}+b_{d\_a}\left(t\right)\right]+w_{a}\left(t\right)+M_{a}\cdot f$$

(8) $$\delta \omega \left(t\right)=\left[b_{c\_g}+b_{d\_g}\left(t\right)\right]+w_{g}\left(t\right)+M_{g}\cdot \omega +G_{g}\cdot f$$

Here, ***b*** is the residual bias of the inertial sensors in the GNSS/INS integrated system after the GNSS update. The bias generally consists of two parts: the constant part that can be modeled as a random constant (i.e., $b_{c\_a}$ and $b_{c\_g}$) and the bias drift that can be modeled as a first-order Gauss–Markov process; ***w*** is the noise of the accelerometer and gyros, which is usually modeled as Gaussian white noise; ***M*** is a 3 × 3 dimensional matrix, which represents the combination of scale-factor error and cross-coupling error, and ***G*** is the g-sensitivity error of the gyroscope; both of them are modeled by a random constant.

Then, the detailed modeling of the INS error in the Laplace domain can be derived from Equations (7) and (8):

(9) $$\delta f\left(s\right)=b_{c\_a}\cdot \frac{1}{s}+w_{b\_d,a}\left(s\right)\cdot \frac{1}{s+\tau_{b\_d,a}}+w_{a}\left(s\right)+M_{a}f\cdot \frac{1}{s}$$

(10) $$\delta \omega \left(s\right)=b_{c\_g}\cdot \frac{1}{s}+w_{b\_d,g}\left(s\right)\cdot \frac{1}{s+\tau_{b\_d,g}}+w_{g}\left(s\right)+M_{g}\omega \cdot \frac{1}{s}+G_{g}f\cdot \frac{1}{s}$$

where τ is the reciprocal of correlation time and $w_{b}$ is the driving noise of the Gauss–Markov process.

### 2.3. Modeling of the Tracking Error in the GNSS/INS Deep Integration

Without loss of generality, take the north direction movement and MEMS INS case as an example; the error propagation models are shown as follows. Based on the same method, the error propagation models of MEMS INS under other directions and tactical-grade INS can be achieved. Substituting Equation (9) into Equation (5), the relationship between the north velocity error and each specific error source can be firstly established:

(11) $$\delta v_{N\_MEMS}(s)=\frac{1}{s}\delta v_{N}(0)-\frac{f_{D}}{s^{2}}\phi_{pitch}\left(0\right)+\frac{f_{E}}{s^{2}}\phi_{yaw}\left(0\right)+\frac{1}{s}\delta f_{N}(s)+\frac{f_{D}}{s^{2}}\delta \omega_{E}(s)-\frac{f_{E}}{s^{2}}\delta \omega_{D}\left(s\right)$$

where

(12) $$\left\{ \begin{array}{l} \delta f_{N}\left(s\right)=\frac{1}{s}b_{c\_a_{N}}+\frac{w_{b\_d,a_{N}}\left(s\right)}{s+\tau_{b\_d,a_{N}}}+w_{a_{N}}\left(s\right)+\frac{1}{s}\left(S_{a,N}f_{N}+M_{a,NE}f_{E}+M_{a,ND}f_{D}\right)\\ \delta \omega_{E}(s)=\frac{1}{s}b_{c\_g_{E}}+\frac{w_{b\_d,g_{E}}\left(s\right)}{s+\tau_{b\_d,g_{E}}}+w_{g_{E}}\left(s\right)+\frac{1}{s}\left(G_{g,EN}f_{N}+G_{g,EE}f_{E}+G_{g,ED}f_{D}\right)\\ \delta \omega_{D}(s)=\frac{1}{s}b_{c\_g_{D}}+\frac{w_{b\_d,g_{D}}\left(s\right)}{s+\tau_{b\_d,g_{D}}}+w_{g_{D}}\left(s\right)+\frac{1}{s}\left(G_{g,DN}f_{N}+G_{g,DE}f_{E}+G_{g,DD}f_{D}\right) \end{array}\right.$$

According to previous research, the inertial aiding information can be induced into the tracking loop as a feed-forward branch in the GNSS/INS deep integration. The Doppler frequency of the carrier signal can be estimated simply by the velocity of the receiver relative to the satellite, projected onto the line of sight (LOS) direction. In order to analyze the worst impact of the maneuver-independent velocity errors on the receiver tracking loop, it is assumed that the satellite is right at the north direction of the receiver (i.e., the maximum projection of the error) [20]. Based on Equation (11), the Doppler aiding information error caused by the INS error sources in high dynamics (i.e., uniform linear acceleration motion) can be presented as:

(13) $$\delta f_{MEMS\_\delta v_{N}}\left(s\right)=\frac{2\pi }{\lambda_{L}}\delta v_{N\_MEMS}(s)\;=\frac{2\pi }{\lambda_{L}}\left[\frac{1}{s}\delta v_{N}(0)-\frac{f_{D}}{s^{2}}\phi_{pitch}\left(0\right)+\frac{f_{E}}{s^{2}}\phi_{yaw}\left(0\right)+\frac{1}{s}\delta f_{N}(s)+\frac{f_{D}}{s^{2}}\delta \omega_{E}(s)-\frac{f_{E}}{s^{2}}\delta \omega_{D}\left(s\right)\right]$$

where $\lambda_{L}$ is the wavelength of the GNSS carrier. Then, the tracking error caused by INS errors will be [20,27]:

(14) $$\delta \theta_{MEMS\_\delta v_{N}}\left(s\right)=-\frac{1}{s}\delta f_{MEMS\_\delta v_{N}}\left(s\right)\cdot \left[1-H\left(s\right)\right]$$

Additionally, *H*(s) is the system transfer function of the receiver tracking loop in the Laplace domain, and taking the 2nd-order tracking loop as an example, it can be described as follows [3,4]:

(15) $$H(s)=\frac{2\xi \omega_{n}s+\omega_{n}^{2}}{s^{2}+2\xi \omega_{n}s+\omega_{n}^{2}}$$

where $\omega_{n}$ is the natural radian frequency of the loop filter and *ξ* is the damping factor. For analysis convenience, the damping factor *ξ* is set to a typical value (e.g., *ξ* = 1).

Substituting the INS-aided information errors in the Laplace domain (i.e., Equation (13)) into Equation (14), the relation between the carrier phase tracking errors and INS errors sources can be expressed as follows:

(16) $$\begin{array}{cc} \delta \theta_{MEMS\_\delta v_{N}}\left(s\right)= & -\frac{2\pi }{\lambda_{L}}\cdot \frac{s}{{\left(s+\omega_{n}\right)}^{2}}[\frac{1}{s}\delta v_{N}(0)-\frac{f_{D}}{s^{2}}\phi_{pitch}\left(0\right)+\frac{f_{E}}{s^{2}}\phi_{yaw}\left(0\right)\\ & +\frac{1}{s}\delta f_{N}(s)+\frac{f_{D}}{s^{2}}\delta \omega_{E}(s)-\frac{f_{E}}{s^{2}}\delta \omega_{D}\left(s\right)] \end{array}$$

where the detailed models of accelerometer error $\delta f_{*}(s)$ and the gyro error $\delta \omega_{*}\left(s\right)$ are shown in Equation (12).

Equation (16) indicates that the main error sources, affecting the tracking loop performance in high dynamics, are INS initial error and inertial sensor error. According to the previous description, the error sources can be divided into non-maneuver-dependent error terms and maneuver independent error terms. Based on Equations (12) and (16), the error propagation models of non-maneuver-dependent error and maneuver independent error will be presented respectively as follows.

#### 2.3.1. Error Propagation Models of Non-Maneuver-Dependent Error

According to Figure 2, the non-maneuver-dependent errors mainly include the INS initial errors, the bias and the noise of inertial sensors. The error propagation models of non-maneuver-dependent error are presented as follows.

(1) Tracking errors caused by the initial velocity error in the north direction $\delta v_{N}(0)$:
  (17) $$\delta \theta_{MEMS\_\delta v_{N\_}\delta v_{N}\left(0\right)}\left(s\right)=-\frac{2\pi }{\lambda_{L}}\cdot \frac{1}{{\left(s+\omega_{n}\right)}^{2}}\cdot \delta v_{N}(0)$$
(2) Tracking errors caused by initial pitch angle error $\phi_{pitch}\left(0\right)$:
  (18) $$\delta \theta_{MEMS\_\delta v_{N}\_\phi_{pitch}\left(0\right)}\left(s\right)=\frac{2\pi f_{D}}{\lambda_{L}}\cdot \frac{1}{s{\left(s+\omega_{n}\right)}^{2}}\cdot \phi_{pitch}\left(0\right)$$
(3) Tracking errors caused by initial yaw angle error $\phi_{yaw}\left(0\right)$:
  (19) $$\delta \theta_{MEMS\_\delta v_{N}\_\phi_{yaw}}\left(s\right)=-\frac{2\pi f_{E}}{\lambda_{L}}\cdot \frac{1}{s{\left(s+\omega_{n}\right)}^{2}}\cdot \phi_{yaw}\left(0\right)$$
(4) Tracking errors caused by the bias constant of the accelerometer in the north direction $b_{c\_a_{N}}$:
  (20) $$\delta \theta_{MEMS\_\delta v_{N}\_b_{c\_a_{N}}}\left(s\right)=-\frac{2\pi }{\lambda_{L}}\cdot \frac{1}{s{\left(s+\omega_{n}\right)}^{2}}\cdot b_{c\_a_{N}}$$
(5) Tracking errors caused by the bias constant of gyro in the east direction $b_{c\_g_{E}}$:
  (21) $$\delta \theta_{MEMS\_\delta v_{N}\_b_{c\_g_{E}}}\left(s\right)=-\frac{2\pi f_{D}}{\lambda_{L}}\cdot \frac{1}{s^{2}{\left(s+\omega_{n}\right)}^{2}}\cdot b_{c\_g_{E}}$$
(6) Tracking errors caused by the bias constant of the yaw gyro $b_{c\_g_{D}}$:
  (22) $$\delta \theta_{MEMS\_\delta v_{N}\_b_{c\_g_{D}}}\left(s\right)=\frac{2\pi f_{E}}{\lambda_{L}}\cdot \frac{1}{s^{2}{\left(s+\omega_{n}\right)}^{2}}\cdot b_{c\_g_{D}}$$
(7) Error propagation of the bias drift of the accelerometer in the north direction $w_{b\_d,a_{N}}\left(s\right)$:
  (23) $$H_{MEMS\_\delta v_{N}\_w_{b\_d,a_{N}}}\left(s\right)=\frac{\delta \theta_{MEMS\_\delta v_{N}\_w_{b\_d,a_{N}}}\left(s\right)}{w_{b\_d,a_{N}}\left(s\right)}=-\frac{2\pi }{\lambda_{L}}\cdot \frac{1}{\left(s+\tau_{b\_d,a_{N}}\right){\left(s+\omega_{n}\right)}^{2}}$$
(8) Error propagation of the bias drift of the gyro in the east direction $w_{b\_d,g_{E}}\left(s\right)$:
  (24) $$H_{MEMS\_\delta v_{N}\_w_{b\_d,g_{E}}}\left(s\right)=\frac{\delta \theta_{MEMS\_\delta v_{N}\_w_{b\_d,g_{E}}}\left(s\right)}{w_{b\_d,g_{E}}\left(s\right)}=-\frac{2\pi f_{D}}{\lambda_{L}}\cdot \frac{1}{s\left(s+\tau_{b\_d,g_{E}}\right){\left(s+\omega_{n}\right)}^{2}}$$
(9) Error propagation of the bias drift of the yaw gyro $w_{b\_d,g_{D}}\left(s\right)$:
  (25) $$H_{MEMS\_\delta v_{N}\_w_{b\_d,g_{D}}}\left(s\right)=\frac{\delta \theta_{MEMS\_\delta v_{N}\_w_{b\_d,g_{D}}}\left(s\right)}{w_{b\_d,g_{D}}\left(s\right)}=\frac{2\pi f_{E}}{\lambda_{L}}\cdot \frac{1}{s\left(s+\tau_{b\_d,g_{D}}\right){\left(s+\omega_{n}\right)}^{2}}$$
(10) Error propagation of the noise of the accelerometer in the north direction $w_{a_{N}}\left(s\right)$:
  (26) $$H_{MEMS\_\delta v_{N}\_w_{a_{N}}}\left(s\right)=\frac{\delta \theta_{MEMS\_\delta v_{N}\_w_{a_{N}}}\left(s\right)}{w_{a_{N}}\left(s\right)}=-\frac{2\pi }{\lambda_{L}}\cdot \frac{1}{{\left(s+\omega_{n}\right)}^{2}}$$
(11) Error propagation of the noise of the gyro in the east direction $w_{g_{E}}\left(s\right)$:
  (27) $$H_{MEMS\_\delta v_{N}\_w_{g_{E}}}\left(s\right)=\frac{\delta \theta_{MEMS\_\delta v_{N}\_w_{g_{E}}}\left(s\right)}{w_{g_{E}}\left(s\right)}=-\frac{2\pi f_{D}}{\lambda_{L}}\cdot \frac{1}{s{\left(s+\omega_{n}\right)}^{2}}$$
(12) Error propagation of the noise of the yaw gyro $w_{g_{D}}\left(s\right)$:
  (28) $$H_{MEMS\_\delta v_{N}\_w_{g_{D}}}\left(s\right)=\frac{\delta \theta_{MEMS\_\delta v_{N}\_w_{g_{D}}}\left(s\right)}{w_{g_{D}}\left(s\right)}=\frac{2\pi f_{E}}{\lambda_{L}}\cdot \frac{1}{s{\left(s+\omega_{n}\right)}^{2}}$$

#### 2.3.2. Error Propagation Models of Maneuver-Dependent Error

According to Figure 2, the maneuver-dependent errors consist of the scale-factor error, cross-coupling error and g-sensitivity error of inertial sensors. The error propagation models of maneuver-dependent error are presented as follows.

(1) Tracking errors caused by the scale-factor error of the accelerometer in the north direction $S_{a,N}$:
  (29) $$\delta \theta_{MEMS\_\delta v_{N}\_S_{a,N}}\left(s\right)=-\frac{2\pi f_{N}}{\lambda_{L}}\cdot \frac{1}{s{\left(s+\omega_{n}\right)}^{2}}\cdot S_{a,N}$$
(2) Tracking errors caused by the cross-coupling error of the accelerometer in the north direction $M_{a,N}$:
  (30) $$\delta \theta_{MEMS\_\delta v_{N}\_M_{a,N}}\left(s\right)=-\frac{2\pi \left(M_{a,NE}f_{E}+M_{a,ND}f_{D}\right)}{\lambda_{L}}\cdot \frac{1}{s{\left(s+\omega_{n}\right)}^{2}}$$
(3) Tracking errors caused by the g-sensitivity error of the gyro in the east direction $G_{g,E}$:
  (31) $$\delta \theta_{MEMS\_\delta v_{N}\_G_{g,E}}\left(s\right)=-\frac{2\pi f_{D}}{\lambda_{L}}\cdot \frac{1}{s^{2}{\left(s+\omega_{n}\right)}^{2}}\cdot \left(G_{g,EN}f_{N}+G_{g,EE}f_{E}+G_{g,ED}f_{D}\right)$$
(4) Tracking errors caused by the g-sensitivity error of the yaw gyro $G_{g,D}$:
  (32) $$\delta \theta_{MEMS\_\delta v_{N}\_G_{g,D}}\left(s\right)=\frac{2\pi f_{E}}{\lambda_{L}}\cdot \frac{1}{s^{2}{\left(s+\omega_{n}\right)}^{2}}\cdot \left(G_{g,DN}f_{N}+G_{g,DE}f_{E}+G_{g,DD}f_{D}\right)$$

The time domain response of the random constant errors (i.e., Equations (17)–(22) and (29)–(32)) can be expressed by the inverse Laplace transform directly:

(33) $$\left\{ \begin{array}{l} \delta \theta_{MEMS\_\delta v_{N\_}\delta v_{N}\left(0\right)}\left(t\right)=-\frac{2\pi \delta v_{N}(0)}{\lambda_{L}}\cdot te^{-\omega_{n}t}\\ \delta \theta_{MEMS\_\delta v_{N}\_\phi_{pitch}\left(0\right)}\left(t\right)=\frac{2\pi f_{D}}{\lambda_{L}}\phi_{pitch}\left(0\right)\cdot \left(-\frac{1}{\omega_{n}}te^{-\omega_{n}t}-\frac{1}{\omega_{n}^{2}}e^{-\omega_{n}t}+\frac{1}{\omega_{n}^{2}}\right)\\ \delta \theta_{MEMS\_\delta v_{N}\_\phi_{yaw}\left(0\right)}\left(t\right)=-\frac{2\pi f_{E}}{\lambda_{L}}\phi_{yaw}\left(0\right)\cdot \left(-\frac{1}{\omega_{n}}te^{-\omega_{n}t}-\frac{1}{\omega_{n}^{2}}e^{-\omega_{n}t}+\frac{1}{\omega_{n}^{2}}\right)\\ \delta \theta_{MEMS\_\delta v_{N}\_b_{c\_a_{N}}}\left(t\right)=-\frac{2\pi b_{c\_a_{N}}}{\lambda_{L}}\left(-\frac{1}{\omega_{n}}te^{-\omega_{n}t}-\frac{1}{\omega_{n}^{2}}e^{-\omega_{n}t}+\frac{1}{\omega_{n}^{2}}\right)\\ \delta \theta_{MEMS\_\delta v_{N}\_b_{c\_g_{E}}}\left(t\right)=-\frac{2\pi f_{D}b_{c\_g_{E}}}{\lambda_{L}}\left(\frac{1}{\omega_{n}^{2}}te^{-\omega_{n}t}+\frac{1}{\omega_{n}^{2}}t+\frac{2}{\omega_{n}^{3}}e^{-\omega_{n}t}-\frac{2}{\omega_{n}^{3}}\right)\\ \delta \theta_{MEMS\_\delta v_{N}\_b_{c\_g_{D}}}\left(t\right)=\frac{2\pi f_{E}b_{c\_g_{D}}}{\lambda_{L}}\left(\frac{1}{\omega_{n}^{2}}te^{-\omega_{n}t}+\frac{1}{\omega_{n}^{2}}t+\frac{2}{\omega_{n}^{3}}e^{-\omega_{n}t}-\frac{2}{\omega_{n}^{3}}\right)\\ \delta \theta_{MEMS\_\delta v_{N}\_S_{a,N}}\left(t\right)=-\frac{2\pi S_{a,N}f_{N}}{\lambda_{L}}\cdot \left(-\frac{1}{\omega_{n}}te^{-\omega_{n}t}-\frac{1}{\omega_{n}^{2}}e^{-\omega_{n}t}+\frac{1}{\omega_{n}^{2}}\right)\\ \delta \theta_{MEMS\_\delta v_{N}\_M_{a,N}}\left(t\right)=-\frac{2\pi \left(M_{a,NE}f_{E}+M_{a,ND}f_{D}\right)}{\lambda_{L}}\cdot \left(-\frac{1}{\omega_{n}}te^{-\omega_{n}t}-\frac{1}{\omega_{n}^{2}}e^{-\omega_{n}t}+\frac{1}{\omega_{n}^{2}}\right)\\ \delta \theta_{MEMS\_\delta v_{N}\_G_{g,E}}\left(t\right)=-\frac{2\pi f_{D}\left(G_{g,EN}f_{N}+G_{g,EE}f_{E}+G_{g,ED}f_{D}\right)}{\lambda_{L}}\left(\frac{1}{\omega_{n}^{2}}te^{-\omega_{n}t}+\frac{1}{\omega_{n}^{2}}t+\frac{2}{\omega_{n}^{3}}e^{-\omega_{n}t}-\frac{2}{\omega_{n}^{3}}\right)\\ \delta \theta_{MEMS\_\delta v_{N}\_G_{g,D}}\left(t\right)=\frac{2\pi f_{E}\left(G_{g,DN}f_{N}+G_{g,DE}f_{E}+G_{g,DD}f_{D}\right)}{\lambda_{L}}\left(\frac{1}{\omega_{n}^{2}}te^{-\omega_{n}t}+\frac{1}{\omega_{n}^{2}}t+\frac{2}{\omega_{n}^{3}}e^{-\omega_{n}t}-\frac{2}{\omega_{n}^{3}}\right) \end{array}\right.$$

The error components (i.e., Equations (23)–(28)) driven by white noise are too complicated to get their time domain expression by the inverse Laplace transform. Hence, Monte Carlo simulations [28] or the convolution integral method [29] can be used to analyze their time domain response based on the transfer functions. Quantitative analysis of INS error transfer, which represents the quality of INS-aided information in high dynamics, can be achieved based on the proposed models above.

## 3. Quantitative Analysis of INS Error Transfer in the Deep Integration in High Dynamics

**Figure 4:**
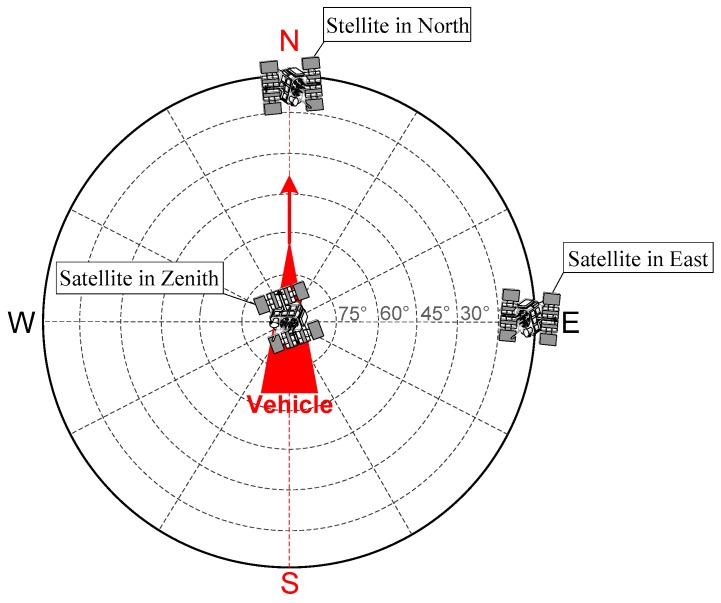
Vehicle in high dynamic uniform acceleration linear motion along the north direction.

With the assumption that the vehicle is in uniform high dynamic linear acceleration motion along the north direction (e.g., $f_{N}=100g$, $f_{E}=0$, $f_{D}=-g$, 1000 m/s), this section presents the quantitative analysis of the carrier phase tracking errors caused by INS error sources including satellites from different directions (i.e., north, east and zenith), as shown in Figure 4. 

Before the quantitative analysis, representative MEMS and tactical INS [30,31] specifications of the error sources are listed in Table 1 and Table 2, and the sources of the parameters are described below:

(a) The parameters are obtained based on real GNSS/INS data processing and the representative IMU specifications; and the GNSS measurements are single point positioning results;
(b) The bias constants are the statistics of the standard deviation of the bias estimation right after the GNSS update;
(c) The initial errors are the statistics of the navigation errors right after the GNSS update;
(d) The other parameters are set according to the real data process parameters.

Without loss of generality, take GPS L1 (i.e., wavelength $\lambda_{L}$ = 0.19 m) as an example: substituting the parameters into the time response models and the Monte Carlo simulations, the quantitative maneuver independent errors of all error sources can be achieved. In the Monte Carlo simulations of the white noise-driven error sources, more than a thousand samples are simulated based on the parameters of different grades of INS, and the statistical results are presented. The analysis results of satellites in the north and east directions and the zenith are shown as follows.

### 3.1. Analysis of the North Satellite Tracking Error

**Figure 5:**
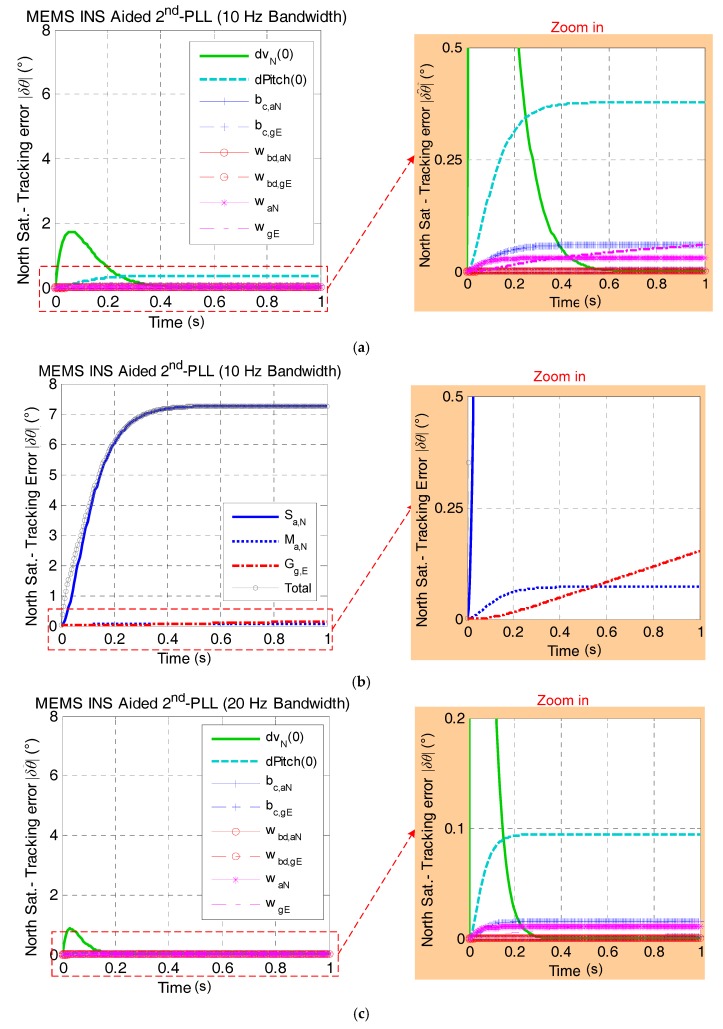

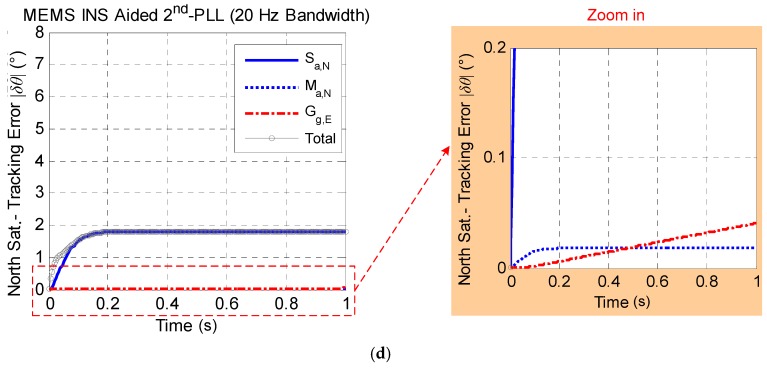
The north satellite tracking error caused by MEMS INS error sources under the assumption of linear high dynamics along the north direction. (**a**) Effect of non-maneuver-dependent error with a 10-Hz bandwidth; (**b**) effect of maneuver-dependent error with a 10-Hz bandwidth; (**c**) effect of non-maneuver-dependent error with a 20-Hz bandwidth; (**d**) effect of maneuver-dependent error with a 20-Hz bandwidth. PLL, phase-locked loop. (* Here, Sat. is the abbreviation of satellite.)

Figure 5 presents the north satellite carrier phase tracking error caused by MEMS INS error sources with bandwidths of 10 Hz and 20 Hz respectively in one second (i.e., the typical GNSS update interval). It is shown that the maximum value of the tracking error in total is less than eight degrees with a bandwidth of 10 Hz and less than two degrees with a bandwidth of 20 Hz. Compared with the tracking threshold (e.g., 45 degrees) [1,2], the impact of the MEMS INS error sources is small enough to be tolerated by the receiver tracking loop in the GNSS/INS deep integration.

Figure 5 also indicates that the major non-maneuver-dependent error sources, which affect the carrier phase tracking accuracy in high dynamics, are the initial velocity errors of the north direction $\delta v_{N}\left(0\right)$ and initial pitch error $\phi_{pitch}\left(0\right)$ by comparing Figure 5a,c. Additionally, Figure 5b,d indicates that the scale-factor error $S_{a,N}$ is the main factor of the tracking error among all of the maneuver-dependent error sources. In general, Figure 5 shows that the main error source that affects the north satellite carrier phase tracking accuracy is the scale-factor error of the north accelerometer. It basically determines the trends and peak value of the tracking error. Figure 5 also shows that the tracking error is smaller and converges faster with the loop bandwidth increasing.

**Figure 6:**
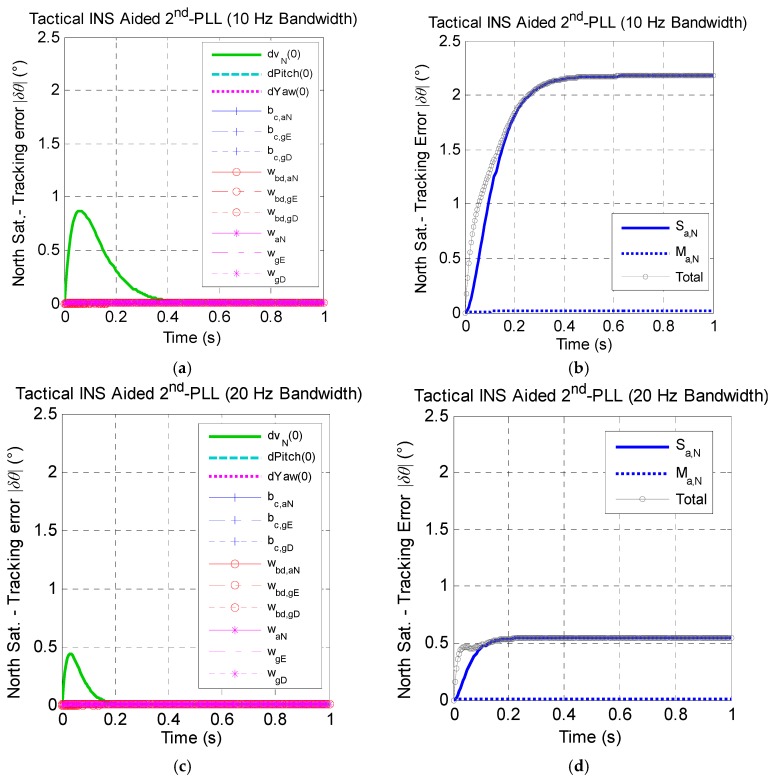
The north satellite tracking error caused by tactical INS error sources under the assumption of linear high dynamics along the north direction. (**a**) Effect of non-maneuver-dependent error with a 10-Hz bandwidth; (**b**) effect of maneuver-dependent error with a 10-Hz bandwidth; (**c**) effect of non-maneuver-dependent error with a 20-Hz bandwidth; (**d**) effect of maneuver-dependent error with a 20-Hz bandwidth. (* Here, Sat. is the abbreviation of satellite.)

Similarly, Figure 6 presents the effect of tactical-grade INS error sources on the north satellite carrier phase tracking performance with bandwidths of 10 Hz and 20 Hz. It shows that the effect of tactical grade INS error sources has the same trends as the MEMS INS, while the difference lies in: (a) the tracking error caused by tactical-grade INS error sources being smaller than the MEMS INS; (b) the effect of the initial pitch error being negligible.

### 3.2. Analysis of the East Satellite Tracking Error

**Figure 7:**
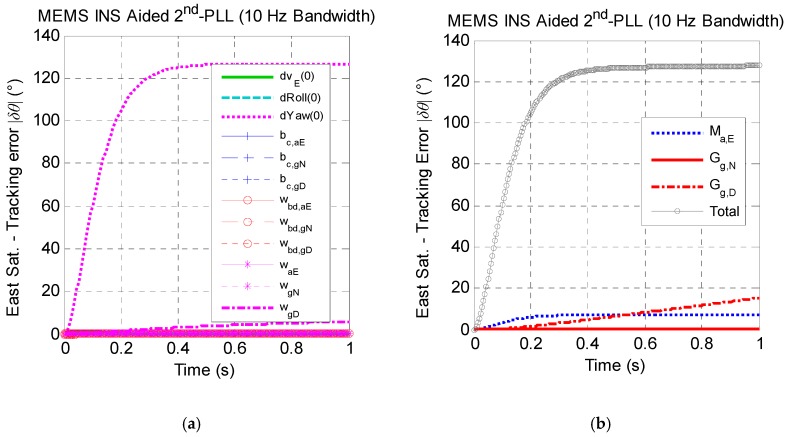

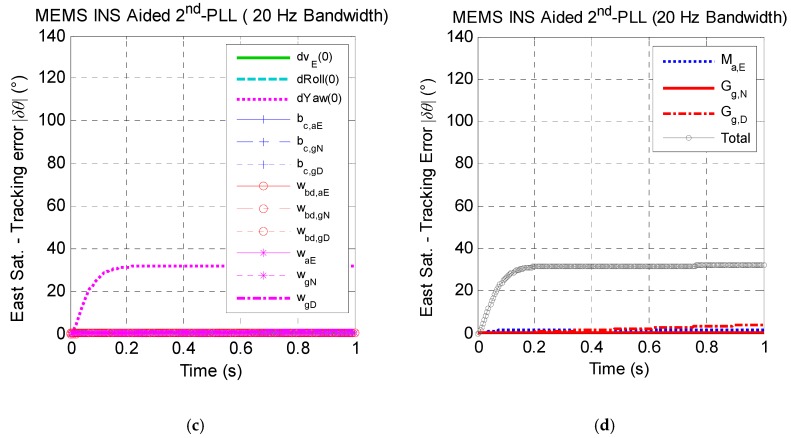
The east satellite tracking error caused by MEMS INS error sources under the assumption of linear high dynamics along the north direction. (**a**) Effect of non-maneuver-dependent error with a 10-Hz bandwidth; (**b**) effect of maneuver-dependent error with a 10-Hz bandwidth; (**c**) effect of non-maneuver-dependent error with a 20-Hz bandwidth; (**d**) effect of maneuver-dependent error with a 20-Hz bandwidth. (* Here, Sat. is the abbreviation of satellite.)

Figure 7 presents the east satellite carrier phase tracking error caused by MEMS INS error sources with bandwidths of 10 Hz and 20 Hz respectively within one second. Figure 7b shows that the total carrier phase tracking error of the east satellite caused by MEMS INS error with a bandwidth of 10 Hz is nearly 130 degrees, which is far greater than the tracking threshold. However, Figure 7d presents that the max tracking error with a bandwidth of 20 Hz is less than 35 degrees, which can keep stable the tracking of the carrier phase. The quantitative analysis results above indicate that the reasonable bandwidth setting of the tracking loop should be a major consideration when designing the GNSS/MEMS INS deep integrated system in high dynamics.

Figure 7a,c shows that the major non-maneuver-dependent error source, which affects the carrier phase tracking accuracy, is the initial yaw error $\phi_{yaw}\left(0\right)$. This indicates that the tracking performance is affected by the initial yaw error, which is coupled with the north accelerometer output. Then, when the vehicle is in high dynamics along the north, a great tracking error will be caused by the initial yaw error. On the other hand, Figure 7b,d also shows that the cross-coupling error of east accelerometer $M_{a,E}$ and the g-sensitivity error of the yaw gyro $G_{g,D}$ are the main factors of the tracking error among all of the maneuver-dependent error sources, but the effect of the non-maneuver-dependent errors is greater than that of the maneuver-dependent errors in total by comparing Figure 7a,c and Figure 7b,d.

**Figure 8:**
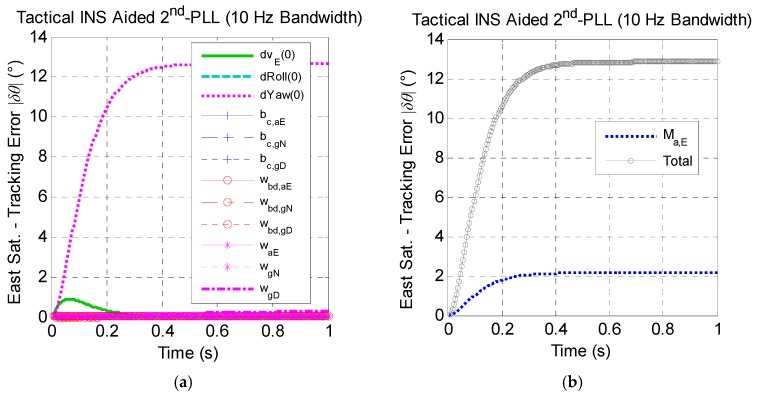

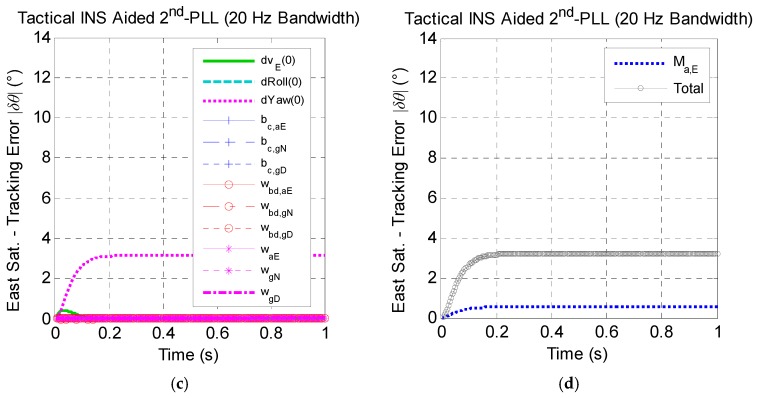
The east satellite tracking error caused by tactical INS error sources under the assumption of linear high dynamics along the north direction. (**a**) Effect of non-maneuver-dependent error with a 10-Hz bandwidth; (**b**) effect of maneuver-dependent error with a 10-Hz bandwidth; (**c**) effect of non-maneuver-dependent error with a 20-Hz bandwidth; (**d**) effect of maneuver-dependent error with a 20-Hz bandwidth. (* Here, Sat. is the abbreviation of satellite.)

Figure 8 presents the effect of tactical-grade INS error sources on the east satellite carrier phase tracking performance. Results show that the effect of tactical-grade INS error sources has the same trends as the MEMS INS, and tracking errors caused by the tactical INS are smaller than the MEMS INS.

### 3.3. Analysis of the Zenith Satellite Tracking Error

**Figure 9:**
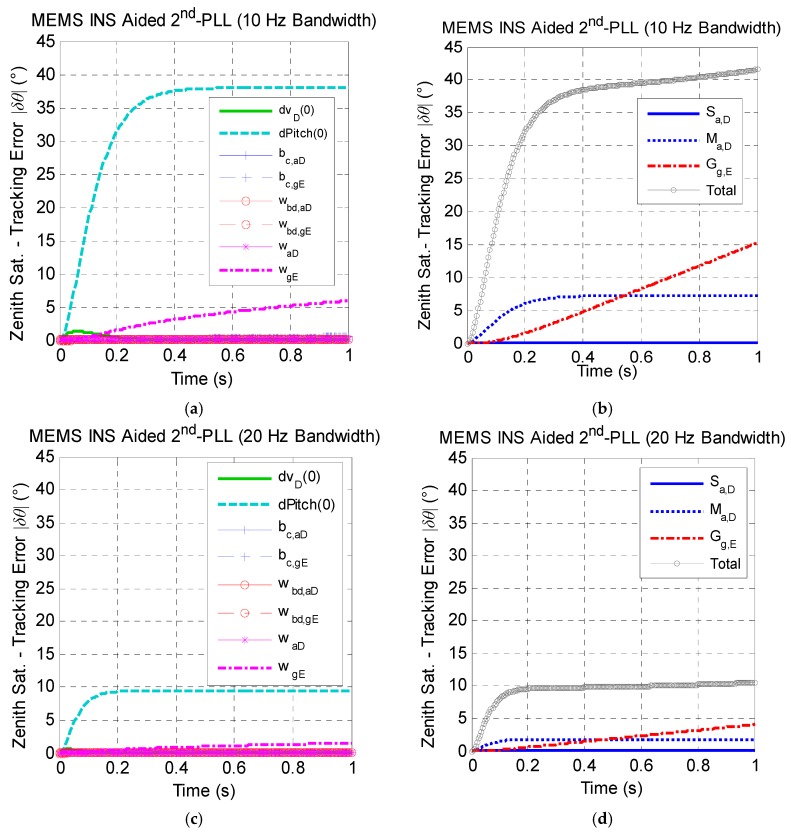
The zenith satellite tracking error caused by MEMS INS error sources under the assumption of linear high dynamics along the north direction. (**a**) Effect of non-maneuver-dependent error with a 10-Hz bandwidth; (**b**) effect of maneuver-dependent error with a 10-Hz bandwidth; (**c**) effect of non-maneuver-dependent error with a 20-Hz bandwidth; (**d**) effect of maneuver-dependent error with a 20-Hz bandwidth. (* Here, Sat. is the abbreviation of satellite.)

Figure 9 gives the zenith satellite carrier phase tracking error caused by MEMS INS error sources with bandwidths of 10 Hz and 20 Hz respectively within one second. As observed in Figure 9b, the zenith satellite tracking error caused by MEMS INS error sources with a bandwidth of 10 Hz is very close to the threshold (e.g., 45 degrees), and the total tracking error curve shows a divergence trend; while the total tracking error with a bandwidth of 20 Hz is only 10 degrees, which is much smaller than the common threshold (see in Figure 9d); and the loop is safe to the keep the carrier phase track steady.

Figure 9a,c shows that the major non-maneuver-dependent error source, which affects the carrier phase tracking accuracy most, is the initial pitch error $\phi_{pitch}\left(0\right)$. Additionally, Figure 9b,d shows that the cross-coupling error of the vertical accelerometer $M_{g,D}$ and the g-sensitivity error of the east gyro $G_{g,E}$ are the main factors of the tracking error among all of the maneuver-dependent error sources. The effect of the non-maneuver-dependent errors is greater than that of maneuver-dependent errors in total by comparing Figure 9a,c and Figure 9b,d.

**Figure 10:**
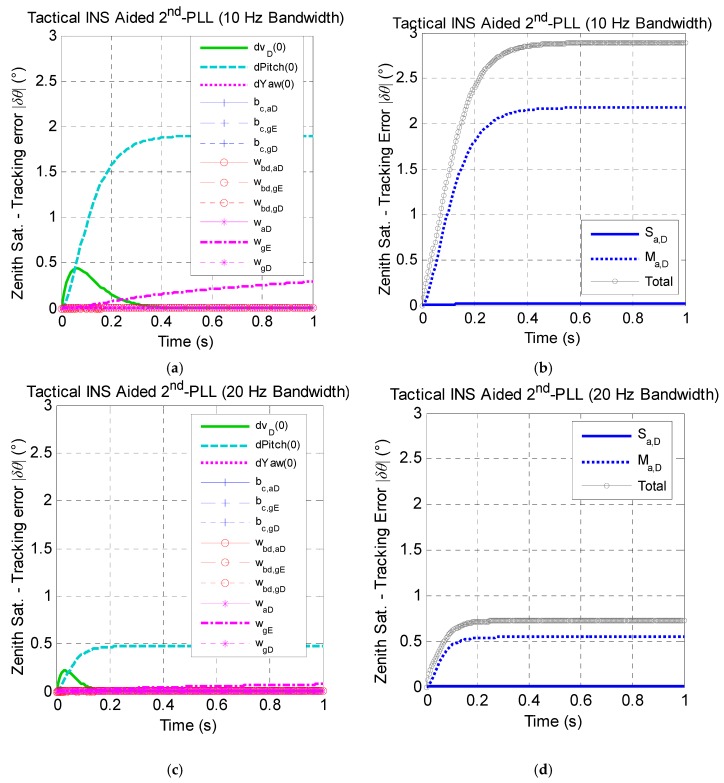
The zenith satellite tracking error caused by tactical INS error sources under the assumption of linear high dynamics along the north direction. (**a**) Effect of non-maneuver-dependent error with a 10-Hz bandwidth; (**b**) effect of maneuver-dependent error with a 10-Hz bandwidth; (**c**) effect of non-maneuver-dependent error with a 20-Hz bandwidth; (**d**) effect of maneuver-dependent error with a 20-Hz bandwidth. (* Here, Sat. is the abbreviation of satellite.)

Figure 10 presents the effect of tactical-grade INS error sources on the east satellite carrier phase tracking performance. Results show that the effect of tactical-grade INS error sources has the same trends with the MEMS INS. Figure 10 also presents that the initial pitch error and the cross-coupling error of the vertical accelerometer of the tactical INS have nearly the same effect on the carrier phase tracking performance, and the effect of the initial velocity error and gyro noise cannot be ignored.

Based on the principles mentioned above, quantitative analysis of the carrier phase tracking errors caused by INS error sources including satellites from different directions (i.e., north, east and zenith) can be achieved by assuming that the vehicle is in uniform high dynamic linear acceleration motion along the east direction (i.e., $f_{N}=0$, $f_{E}=100g$, $f_{D}=-g$ ) or the vertical direction (i.e., $f_{N}=0$, $f_{E}=0$, $f_{D}=-101g$). Then, the main factors of the INS error sources that affect the carrier phase tracking performance in the GNSS/INS deep integration can be concluded from Table 3. According to the analysis above, the results point out that:

(1) The effects of INS error sources are related to the motion direction and the satellite position. The effect of INS error sources on the tracking performance of the east satellite is relatively great when the vehicle is in the uniform high dynamic linear acceleration motion along the north direction; and the effect of INS error sources on the tracking performance of the north satellite is relatively great when the vehicle moves along the east direction.
(2) The main factors of INS error sources vary according to the relative position of the satellite and vehicle. When the satellite is in the direction of the vehicle motion, the main error source that affects the carrier phase tracking performance is the accelerometer scale factor error of the vehicle motion direction; when the satellite is in the orthogonal direction of the vehicle motion, the main error sources that affect the carrier phase tracking performance are the initial attitude error and the cross-coupling error of the LOS direction.
(3) The initial attitude error, which is always coupled with the accelerometer output, will cause a great effect on the carrier phase tracking performance when the vehicle is in linear high dynamics.
(4) It should be noted that the effect of gyro white noise and g-sensitivity error on the tracking performance continues to diverge over time.
(5) Results indicate that the MEMS INS can be used in the GNSS/INS deep integration by aiding the carrier phase tracking loop with the proper loop bandwidth in high dynamics.
(6) The tracking error is smaller and converges faster with the loop bandwidth increasing.

## 4. Conclusions

This paper presented a quantitative analysis of the impacts of the aiding information from different grades of IMUs by developing the INS error propagation models of GNSS/INS deep integrated systems in high dynamics. Under the assumption of uniform linear acceleration motion (100 g), it establishes the connections between all INS error sources and carrier phase tracking errors by the INS error dynamic equations and the INS-aided PLL model. Then, it quantitatively analyzed the effects of the INS maneuver-dependent and non-maneuver-dependent error sources on carrier phase tracking performance respectively, when the receiver moves along the north, east and vertical directions with large accelerations.

The analysis shows that: the major error sources, which affect the carrier phase tracking accuracy in high dynamics, are the initial attitude errors, accelerometer scale factors, gyro noise and g-sensitivity errors. The initial attitude errors usually take effect with the receiver acceleration to impact tracking performance, which can easily cause the failure of signal tracking. Besides, the main error factors vary with the receiver motion direction and the relative position of the receiver and satellites. The analysis results also indicate that even the low-end MEMS IMU has the ability to provide the aiding information with sufficient quality for the GNSS signal carrier phase tracking in high dynamics, and the higher grade (e.g., tactical grade) IMUs have smaller drift errors. The quantitative analysis results can guide the selection of the inertial sensors and the implementation of the GNSS/INS deep integration for high dynamic applications.

It should be noted that the analysis in this paper focused on the short-term (i.e., 1 s) performance of the INS aiding for the GNSS signal tracking with continuous GNSS update. It does not apply to the long-term cases, such as GNSS outages and INS-aided re-acquisition.

## Figures and Tables

**Table 1:** Non-maneuver-dependent error specifications of different grades of INS.

Characteristics		Symbols	MEMS INS	Tactical INS
Gyro	bias constant *	$b_{c\_g}$	15°/h	0.1°/h
	mean squared value of bias drift (1σ)	$\sigma_{b\_d,g}$	100°/h	0.1°/h
	correlation time	$T_{b\_d,g}$	600 s	10,800 s
	power spectral density (PSD) of white-noise	$P_{w_{g}}$	(3°/h^−1/2^)^2^	(0.15°/h^−1/2^)^2^
Accelerometer	bias constant *	$b_{c\_a}$	800 mGal	50 mGal
	mean squared value of bias drift (1σ)	$\sigma_{b\_d,a}$	2000 mGal	100 mGal
	correlation time	$T_{b\_d,a}$	600 s	10,800 s
	PSD of white-noise	$P_{w_{a}}$	(0.12 m·s^−1^·h^−1/2^)^2^	(0.03 m·s^−1^·h^−1/2^)^2^
Initial Error **	horizontal velocity	$\delta v_{N/E}\left(0\right)$	0.04 m/s	0.025 m/s
	vertical velocity	$\delta v_{D}\left(0\right)$	0.03 m/s	0.015 m/s
	roll/pitch	$\phi_{roll}\left(0\right)$ $\phi_{pitch}\left(0\right)$	0.30°	0.015°
	yaw	$\phi_{yaw}\left(0\right)$	1.00°	0.100°

* Residual bias errors after GPS update; ** residual navigation errors after GPS update.

**Table 2:** Maneuver-dependent error specifications of different grades of INS.

Characteristics		Symbols	MEMS INS	Tactical INS
Gyro	Scale-factor Error	$S_{g}$	1000 ppm	300 ppm
	Cross-coupling Error	$M_{g}$	1000 ppm	300 ppm
	g-sensitivity Error	$G_{g}$	5°/h·g^−1^	-
Accelerometer	Scale-factor Error	$S_{a}$	1000 ppm	300 ppm
	Cross-coupling Error	$M_{a}$	1000 ppm	300 ppm

**Table 3:** The main error sources affect the tracking performance in linear high dynamics.

Satellite Position	The Direction of Uniform Linear Acceleration Motion		
	North	East	Vertical
**North**	**Accel. Scale-Factor of North**	**Initial Yaw Error**	**Initial Pitch Error**
	Initial velocity error of north Initial pitch error *	Accel. cross-coupling error of north Gyro g-sensitivity error of yaw * Gyro white noise of yaw Initial velocity error of north	Accel. cross-coupling error of north Gyro g-sensitivity error of east * Gyro white noise of north Initial velocity error of north
**East**	**Initial Yaw Error**	**Accel. Scale-Factor of East**	**Initial Roll Error**
	Accel. cross-coupling error of east Gyro g-sensitivity error of yaw * Gyro white noise of Yaw Initial velocity error of east	Initial velocity error of east Initial roll error *	Accel. cross-coupling error of east Gyro g-sensitivity error of north * Gyro white noise of north Initial velocity error of east
**Zenith**	**Initial Pitch Error**	**Initial Roll Error**	**Accel. Scale-Factor of Vertical**
	Accel. cross-coupling error of vertical Gyro g-sensitivity error of east * Gyro white noise of east Initial velocity error of vertical	Accel. Cross-coupling error of vertical Gyro g-sensitivity error of north * Gyro white noise of north Initial velocity error of vertical	Initial velocity error of vertical

* Only in the MEMS INS case; Accel. is the abbreviation of accelerometer.
